# Characterising HIV-Indicator conditions among two nationwide long-term cohorts of people living with HIV in Germany (1999–2023)

**DOI:** 10.1007/s15010-024-02419-2

**Published:** 2024-10-30

**Authors:** Amrei Krings, Christian Kollan, Daniel Schmidt, Barbara Gunsenheimer-Bartmeyer, Frederik Valbert, Anja Neumann, Jürgen Wasem, Georg M. N. Behrens, Markus Bickel, Christoph Boesecke, Stefan Esser, Patrik Dröge, Thomas Ruhnke, Uwe Koppe, the HIV-1 Seroconverter, ClinSurv-HIV study groups, Stefan Esser, Heribert Knechten, Petra Panstrugart, Keikawus Arasteh, Michael Rittweger, Hans Wesselmann, Nikolai Menner, Dirk Schürmann, Marianne Warncke, Ulrich Bohr, Heiko Jessen, Arne B. Jessen, Hubert Schulbin, Sascha Brand, Jan Gumprecht, Beate Weninger, Heribert Hillenbrand, Heiko Karcher, Klaus Fischer, Dietmar Schranz, Mathias Vallée, Jukka Hartikainen, Stephan Grunwald, Jörg A. Claus, Claudia Thomas, Roland Grimm, Sarah Schoor, Christiane Cordes, Reinhold Schröder, Tobias Glaunsinger, Michael Rausch, Thomas Reineke, Gordon Weinberg, Manuel Bruhy, Siegfried Köppe, Peter Kreckel, Andreas Berger, Sinah Lindemann, Norbert H. Brockmeyer, Anja Potthoff, Kathrin van Bremen, Jürgen Rockstroh, Jan-Christian Wasmuth, Svetlana Hass, Martin Hower, Claudia Bachmann, Petra Spornraft-Ragaller, Dieter Teichmann, Björn-Erik Ole Jensen, Falk Hüttig, Cecilie Feind, Pia Schenk-Westkamp, Annette Haberl, Christoph Stephan, Peter Schott, Susanne Usadel, Matthias Müller, Janina Trauth, Alan Chavez-Valladares, Gerd Deutschinoff, Burkhard Kreft, Danica Lange, Olaf Degen, Guido Schäfer, Andreas Plettenberg, Frieder Kuhlendahl, Thore Lorenzen, Dorothea Wiemer, Lavinia Biemann, Axel Adam, Thomas Buhk, Stephan Fenske, Stefan Hansen, Michael Sabranski, Knud Schewe, Christian Hoffmann, Hans-Jürgen Stellbrink, Dennis Radzuweit, Alexander Mainka, Constantin Rickassel, Olaf Degen, Guido Schäfer, Robin Scheiter, Georg Behrens, Matthias Stoll, Steve Gerschmann, Renate Beider, Benjamin T. Schleenvoigt, Mathias W. Pletz, Heinz-August Horst, Silke Trautmann, Ansgar Rieke, Stephan Schneeweiß, Stefan Scholten, Mark Oette, Gerd Fätkenheuer, Jörg Janne Vehreschild, Laura Hamacher, Lennart Nicksch, Peter A. Arbter, Thomas Grünewald, Jeannine Weidemann, Ines Ruck, Bernd Claus, Martin Sprinzl, Peter R. Galle, Matthias P. Ebert, Roger Vogelmann, Johannes Bogner, Ulrike Hellerer, Barbara Sonntag, Oliver Pullen, Antoniya Todorova, Claudia Traidl-Hoffmann, Birgit Mück, Ramona Pauli, Christoph D. Spinner, Jochen Schneider, Birgit Mück, Robert Baumann, Niels Schübel, Christiane Berning, Franz Audebert, Carlos Fritzsche, A Trein, E Schnaitmann, Clemens Roll, Simone Marquardt, Georg Härter, Beate Grüner, Cengiz Güler, Steve Rößler

**Affiliations:** 1 Department of Infectious Disease Epidemiology, Robert Koch Institute, Seestrasse 10, Berlin, Germany; 2 Institute for Healthcare Management and Research, University Duisburg-Essen, Thea-Leymann-Str. 9, Essen, Germany; 3 Department for Rheumatology and Immunology, Hannover Medical School, Carl-Neuberg-Str. 1, Hanover, Germany; 4 German Centre for Infection Research (DZIF), Partner Site Braunschweig-Hannover, Hanover, Germany; 5 Infektiologikum Frankfurt, Stresemannallee 3, Frankfurt, Germany; 6 Department of Medicine I, Bonn University Hospital, Venusberg-Campus 1, Bonn, Germany; 7 Department of Dermatology and Venerology, University Hospital Essen, University Duisburg-Essen, Hufelandstrasse 55, Essen, Germany; 8 AOK Research Institute (WIdO), Rosenthaler Strasse 31, Berlin, Germany; 9 German Centre for Infection Research (DZIF), Bonn, Germany

**Keywords:** HIV, Human immunodeficiency virus, AIDS, Acquired immune deficiency syndrome, Indicator conditions, Germany, Cohort studies

## Abstract

**Background/Objective:**

Information about occurrence and affected groups of symptoms/diagnoses indicative of an HIV infection (so-called HIV indicator conditions; HIV-ICs) is lacking. We analyse HIV-IC incidence, transmission risks and immune status among people living with HIV (PLWH) antiretroviral therapy (ART) naive.

**Methods:**

Diagnoses reported for ART-naive PLWH from two multicentre observational, prospective cohort studies between 1999–2023 were analysed. Incidence rates per 1,000 person-years (PYs) were calculated for the overall study period and time periods defined by ART treatment recommendations. For further description, CD4 counts around HIV-IC diagnosis (+ -30 days) and HIV-transmission routes were collected.

**Results:**

In total 15,940 diagnoses of 18,534 PLWH in Germany were included. Of those 81% were male (median age: 36 years) and 56% reported being men, who have sex with men as the likely HIV-transmission route. Incidence rates varied between the different HIV-ICs. Syphilis had the highest incidence rate (34 per 1,000 PYs; 95% confidence interval [CI] 29–40) for sexually transmitted infections (STIs), hepatitis B was highest for viral hepatitis diagnoses (18 per 1,000 PYs; 95% CI 17–20); according to CDC-classification herpes zoster for HIV-associated diagnoses (22 per 1,000; 95% CI 20–24) and candidiasis for AIDS-defining diagnoses (30 per 1,000 PYs; 95% CI 29–32). Most PLWH with HIV-ICs (hepatitis, HIV-associated diagnoses and AIDS-defining conditions) had CD4 cell counts < 350.

**Conclusion:**

This analysis characterizes HIV-ICs regarding the incidence, HIV-transmission route and patients’ immune status. The results underline the importance of HIV-IC-based screening to detect PLWH with already partially impaired immune status and in need of timely ART initiation.

**Supplementary Information:**

The online version contains supplementary material available at 10.1007/s15010-024-02419-2.

## Background

According to the current WHO strategy, infections with human immunodeficiency virus (HIV), hepatitis B virus, hepatitis C virus, and other sexually transmitted infections (STI) shall be eliminated as a public health threat until 2030 [1, 2]. Therefore, the WHO has defined the so called 95–95-95 targets for HIV, which state that until 2030 95% of people living with HIV (PLWH) know their status, of those 95% are on antiretroviral treatment (ART) and again of those 95% have suppressed viral loads and hence transmission of HIV is not possible [2, 3].

For Germany, modelling data estimate that about 96.700 people live with HIV by the end of 2023, with 2.200 new infections estimated to have occurred during 2023. Of all PLWH, about 92% are diagnosed and know about their HIV infection [4]. Therefore, Germany does not yet reach the global targets for HIV with regards to the proportion of PLWH and knowing about their infection. The same analysis shows that especially among people with HIV transmission through heterosexual transmission or injecting drug use (IDU) a large proportion of people is undiagnosed [4]. This is also reflected in the 33% late diagnoses with advanced immune defect defined as CD4 cell count < 200 CD4 cells/µl or even AIDS (18%) of all HIV diagnoses. While the number of new HIV infections is based on modelling data, HIV diagnoses are notified to the Robert Koch-Institute (RKI) and need to be distinguished from each other. While the number of late HIV diagnoses decreased among men, who have sex with men (MSM) and to a smaller extent among people indicating heterosexual transmission, it has increased among people with IDU-associated transmission during the last years [4]. A multi-centre study found older age, sexual contacts with both sexes as possible route of HIV transmission, being married, and a poor level of knowledge about HIV treatment as factors associated with late HIV presentation in Germany [5]. Overall, early diagnosis is essential not only on an individual level to enable early induced treatment and prevent immune defects, but also to prevent further transmissions [3, 6–8].

Several screening approaches have been recommended and applied in different contexts in order to reach an early HIV diagnosis for PLWH, who are unaware of their status [9–11]. In the European context of low HIV prevalence the highest prevalence is found among MSM, people with injecting drug use (PWID), people who migrated from high-prevalence countries, etc. Therefore, a risk-based testing strategy is recommended [10].

In addition, it was shown that using a testing approach that is based on testing patients with symptoms/diagnoses indicative of an HIV infection (so-called HIV indicator conditions [HIV-ICs]) is cost-effective in low-prevalence countries and has been confirmed for the European context [12, 13]. HIV-ICs are defined in three disease categories and include (i) AIDS-defining conditions among PLWH, (ii) conditions with a HIV prevalence of more than or equal to 0.1% and (iii) conditions that may have significant adverse patient management outcomes, when an HIV infection would not be diagnosed [14]. Based on these results the European Centre for Disease Prevention and Control (ECDC) has added HIV-IC-based testing into their testing recommendation to complement the risk-based testing approach [10]. Although HIV-IC-based testing strategies have been recommended for low-prevalence countries, its implementation is progressing only slowly or lacking in several countries [15]. This may be due to unavailable evidence with regards to the frequency of conditions occurring as well as lacking information on who is affected, for example with regards to the HIV transmission risk or immune status.

The HeLP study ( “HIV-Testempfehlungen in Leitlinien und Praxis”) was set up in order to validate HIV-IC for Germany, screen HIV testing recommendations in the treatment guidelines for the respective conditions and assess the acceptance of HIV testing recommendations among treating physicians in order to improve HIV-IC guided testing [16]. The validation of HIV-ICs is conducted in two parts. The first part analyses healthcare claims data by calculating the HIV prevalence among diagnosed cases for described HIV-ICs [17]. This analysis shows the results of the second part, which intends to further characterise HIV-ICs based on the description of incidence over time and identification of further aspects such as the HIV transmission risk and immune status. We focussed on PLWH ART-naive or in the early phase (first 6 months) of treatment to represent the occurrence of HIV-ICs as close as possible to patients undergoing HIV-IC screening in clinical practice. This analysis will provide additional insights on HIV-ICs and affected groups.

## Methods

### Study design and population

The people included in this analysis are study participants from two HIV cohorts in Germany, the HIV-1 Seroconverter Study and the ClinSurv-HIV Study. Both studies are nationwide, multicentre, long-term observational, prospective cohort studies. The HIV-1 Seroconverter Study was initiated in 1997 and had 87 study centres participating over time and recruits patients with a known or reliably estimated date of HIV-1 seroconversion. The ClinSurv-HIV Study was initiated in 1999 and recruits patients with newly diagnosed or established HIV infections from a total of 15 participating study centres. There is an overlap between the two cohort studies. Further methodological details of the study designs have been published elsewhere [18–21].

In our study population, we included all PLWH observed in these two cohorts from Germany (n = 26.992; see Supplemental Fig. 1). Participants were excluded from this analysis if they had less than 3 months total observation time (n = 1.743) or already received ART before or at the day when entering the cohort studies (n = 6.715). The start of follow-up was the beginning of observation without ART and ended 6 months after ART initiation. Hence, 727 patients were censored after ART initiation.

**Fig. 1:**
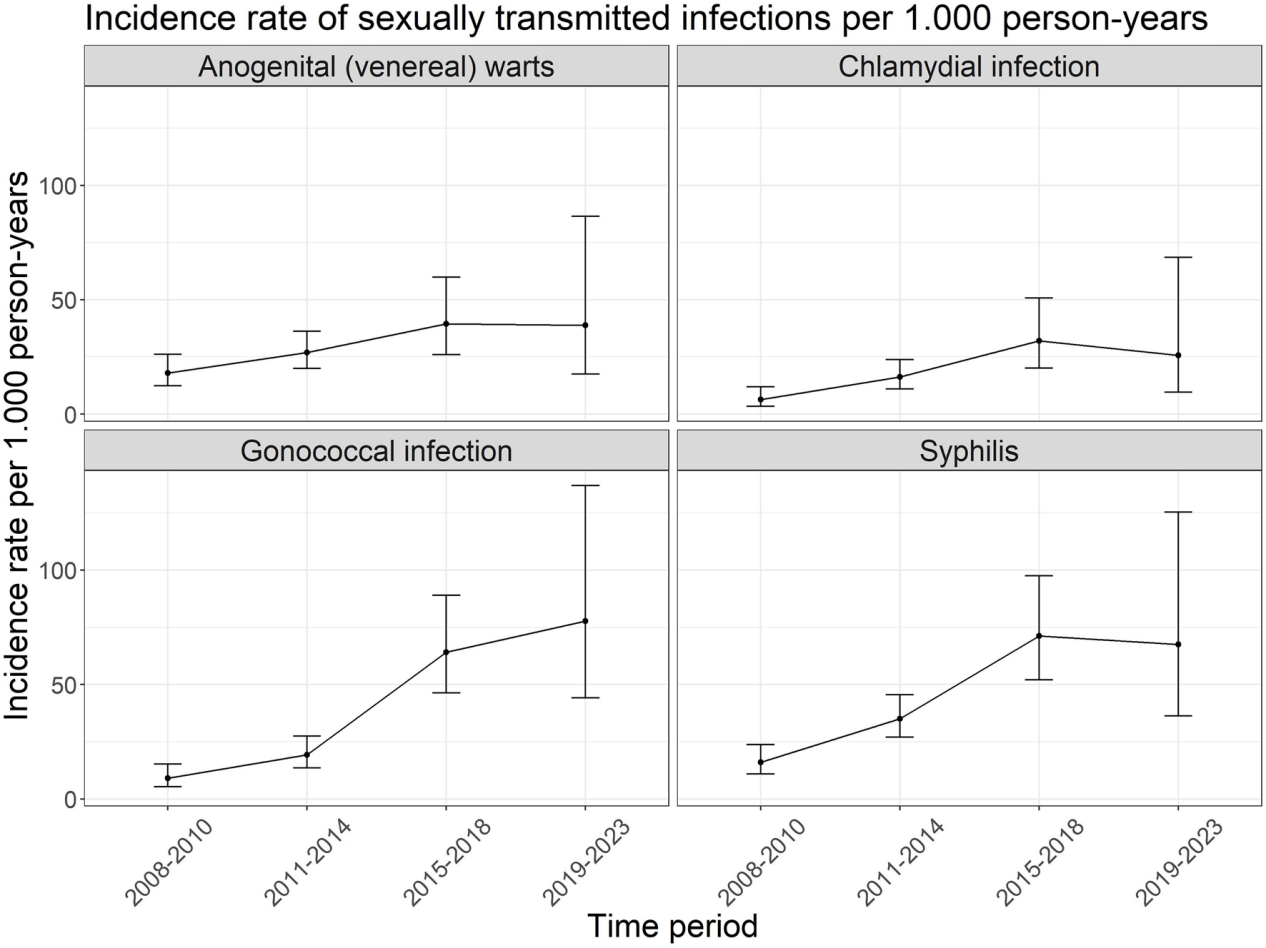
Incidence rates of sexually transmitted infections per 1.000 person-years among HIV-1 Seroconverter cohort participants by time period 2008–2010, 2011–2014, 2015–2018 and 2019–2023

The study period for this analysis was from January 1999 until December 2023, with HIV-1 Seroconverter Study participants contributing for the entire study period and ClinSurv-HIV Study participants contributing until December 2018. The data from the HIV-1 Seroconverter study for 1997–1998 were excluded due to short follow-up during the initiation phase of the cohort. The study period for the analysis of STI was from 2008 to 2023, as not all diagnoses for HIV-ICs were equally collected over the study period, as further described below.

### Data collection

The HIV-1 Seroconverter Study questionnaire contains specific questions with tick boxes for diagnoses classified by the CDC-classification system for HIV infection [22], while the ClinSurv-HIV Study centres are encouraged to report these diagnoses, but are not actively reminded to do so. Additionally, a chapter for STIs was included in the HIV-1 Seroconverter Study questionnaire in 2008. This might lead to an underreporting of diagnoses that may not be primarily perceived as AIDS-defining but rather as associated with HIV or its transmission pathway, such as other STIs, in data collected through the ClinSurv-HIV Study or before 2008. Therefore, the prevalence and incidence analyses of STIs, namely anogenital (venereal) warts, chlamydial, gonococcal and HPV infections, syphilis as well as trichomoniasis were solely based on HIV-1 Seroconverter Study data and only from 2008 onwards. For the analysis of all other HIV-ICs selected for this analysis, data from both cohort studies and the complete study period were used.

### Outcomes

The selection of HIV-ICs for analysis is based on a literature search as published by Valbert et al. [17] and is used in the analysis of healthcare claims data for HIV prevalence among patients with the respective diagnosis and presented in the same manuscript. The following HIV-ICs with the respective ICD-10-GM diagnosis codes were included in the analysis (see Table 1) [23]:

**Table 1:** Diagnostic codes based on ICD-10-GM [23] for selected HIV-ICs used for this analysis

HIV indicator condition	ICD-10 code
Sexually transmitted infections	
Anogenital (venereal) warts	A63.0
Chlamydial infection	A55, A56, A74.9
Gonococcal infection	A54
Papillomavirus as the cause of diseases	B97.7, Z22.8 if textual Diagnosis indicates “HPV”
Syphilis	A50, A51, A52, A53
Trichomoniasis	A59
Viral Hepatitis infections	
Hepatitis A	B15
Hepatitis B	B16, B17.0, B18.0, B18.1
Hepatitis C	B17.1, B18.2
Hepatitis E	B17.2
Viral hepatitis type unknown	B17.8, B18.8, B18.9, B19
HIV-associated diagnoses (CDC-category B)	
Dysplasia of cervix uteri	D06.9, N87
Herpes zoster	B02, B20 with B02.9
Hodgkin lymphoma	C81
Infectious mononucleosis	B27
Malignant neoplasm of anus and anal canal	C21
Malignant neoplasm of bronchus and lung	C34
Oral hairy leukoplakia	K13.2, K13.3, B23 with K13.2
Seborrheic dermatitis	L21
AIDS-defining diagnoses (CDC-category C)	
Abnormal weight loss	R63.4, R64, B22 with R64
Candidiasis	B37, B20 with B37
Herpes simplex infections	A60, B00, B20 with B00.9
Kaposi sarcoma	C46, B21 with C46
Malignant neoplasm of cervix uteri	C53
Non-Hodgkin lymphoma	C82, C83, C84, C85, C86, B21 with C83, B21 with C85
Pneumocystosis	B48.5, B48.5 + J17.2*, B20 with B48.5
Pneumonia	J10.0, J11.0, J12, J13, J14, J15, J16, J17, J18, B20 with J12, B20 with J18
Tuberculosis	A15, A16, A17, A18, A19, B20 with A16.9

*CDC* Centers for Disease Control and Prevention, *ICD* International Statistical Classification of Diseases and Related Health Problems

Diagnoses were included from the time of entering the cohort study until the last available report or until 6 months post ART initiation, in accordance with the assessment of clinical experts. For some study participants (n = 38) laboratory results indicated an HIV RNA viral load below the detection limit (< 50 copies/mL) despite missing information regarding ART. For those participants it was estimated that ART was initiated 3 months prior to the respective laboratory result. We therefore included only diagnoses until 3 months after HIV RNA viral load results below the detection limit and excluded all diagnoses reported after this 3 months’ time period.

While multiple diagnoses were generally plausible for the incidence calculations for some diagnoses, as described in detail below, multiple diagnoses were not considered for the calculation of prevalence.

Multiple diagnoses per study participant were excluded from the incidence calculation if reported for the following HIV-ICs: abnormal weight loss, dysplasia/malignant neoplasm of cervix uteri, hepatitis A, hepatitis B, hepatitis D/E, herpes simplex infections, Hodgkin lymphoma, infectious mononucleosis, Kaposi sarcoma, malignant neoplasm of anus and anal canal, malignant neoplasm of bronchus and lung, non-Hodgkin lymphoma, papillomavirus as the cause of diseases, oral hairy leukoplakia, pneumocystosis, seborrheic dermatitis, tuberculosis. For these HIV-ICs, person time contributed by each study participant was calculated from the first month of participation until the first occurrence of the respective diagnosis.

For HIV-ICs with plausible reinfection or recurrence of the respective condition, multiple diagnoses were included in the incidence calculation. However, a defined time lag was excluded from the analysis after each diagnosis, assuming this time was needed for the condition to be treated. Person time contributed was calculated from the first month until the respective first diagnosis and then after the defined time lag again until the next diagnosis or the last available report. These diagnoses were: anogenital (venereal) warts (time lag: 6 months), candidiasis (time lag: 3 months), chlamydial infection (time lag: 3 months), gonococcal infection (time lag: 3 months), hepatitis C (time lag: 6 months), herpes zoster (time lag: 3 months), pneumonia (no time lag), syphilis (time lag: 6 months for early syphilis and 24 months for late syphilis), trichomoniasis (3 months).

### Statistical analysis

Descriptive statistics (Stata 14, RStudio Version 2023.06.2) were used to investigate sociodemographic and clinical characteristics of the study participants.

Diagnosis prevalence was calculated as the proportion of eligible study participants with the respective HIV-IC among all eligible study participants, as described above. Incidence rates are reported per 1.000 person-years, using monthly data. The prevalence and incidence calculation of dysplasia/malignant neoplasm of cervix uteri was calculated among female participants, only.

Incidence analyses were stratified by the following time periods, based on the different ART treatment recommendations applied in Germany: 1999–2005, 2006–2010, 2011–2014, 2015 onwards. Stratified analyses for STIs, namely anogenital (venereal) warts, chlamydial infection, gonococcal infection, papillomavirus as the cause of diseases, syphilis and trichomoniasis, were conducted from 2008 onwards. As a test for trend a one-step Newton approximation to the log-linear Poisson regression coefficient was computed and the Mantel–Haenszel-type method used to test for significance.

CD4 counts were analysed descriptively, if they were measured within a time interval of 30 days before/after the respective diagnosis. Association between HIV-transmission risk of participants and diagnosis of HIV-IC was tested using Pearson’s chi-squared test. For variables with categories containing less than five observations, Fischer’s exact chi-squared test was used.

### Data protection and ethics

The HIV-1 Seroconverter Study was approved by the Ethics Committee of the Charité University Medicine Berlin (EA2/024/21). Participants of the HIV-1 Seroconverter Study provide their written informed consent to participate. The ClinSurv-HIV Study data (1999–2018) were collected anonymously and in compliance with the German Infection Protection Act (IfSG, 2001). These data did therefore not require written informed consent. Approval for this was granted by the RKI data protection officer and the Federal Commissioner for Data Protection and Freedom of Information.

## Results

In total, 15,940 diagnoses of 18,534 study participants were eligible for the analysis of HIV-ICs in the time period 1999 until 2023 (Table 2). Of those, 14,174 diagnoses were included from the ClinSurv-HIV Study and 1,766 diagnoses from the HIV-1 Seroconverter Study (see Supplemental Fig. 1 for an overview of the excluded patients). The sociodemographic characteristics of eligible study participants overall and stratified by cohort study are shown in Table 2. The majority of participants in both cohort studies were male (81%) and the median age at the beginning of the observation was 36 years (interquartile range [IQR]: 29–44 years). Overall, 56% reported being MSM as the likely route of HIV-transmission, followed by 16% who reported heterosexual contact as the likely HIV-transmission route (Table 2). In the HIV-1 Seroconverter study, we observed a higher percentage of male study participants (94%) with the majority reported being MSM as their likely route of HIV-transmission compared to the ClinSurv-HIV Study. The age difference between the two cohort studies was small.

**Table 2:** Sociodemographic characteristic of included study participants stratified by cohort study

Characteristic	Overall, N = 18,534^1^	ClinSurv-HIV participants, N = 15,489^1^	HIV-1 Seroconverter participants, N = 3,045^1^
Age at observation start	36 (29–44)	37 (30–45)	33 (27–40)
Sex			
Female	3,384 / 18,534 (18%)	3,223 / 15,489 (21%)	161 / 3,045 (5.3%)
Male	15,086 / 18,534 (81%)	12,209 / 15,489 (79%)	2,877 / 3,045 (94%)
Trans-male	57 / 18,534 (0.31%)	50 / 15,489 (0.32%)	7 / 3,045 (0.23%)
Trans-female	7 / 18,534 (0.04%)	7 / 15,489 (0.05%)	0 / 3,045 (0%)
Likely HIV transmission route			
Men, who have sex with men	10,352 / 18,534 (56%)	7,698 / 15,489 (50%)	2,654 / 3,045 (87%)
Injecting drug use	1,318 / 18,534 (7.1%)	1,264 / 15,489 (8.2%)	54 / 3,045 (1.8%)
Heterosexual	2,964 / 18,534 (16%)	2,723 / 15,489 (18%)	241 / 3,045 (7.9%)
High-prevalence country	1,854 / 18,534 (10%)	1,830 / 15,489 (12%)	24 / 3,045 (0.79%)
Uknown	1,819 / 18,534 (9.8%)	1,765 / 15,489 (11%)	54 / 3,045 (1.8%)
Oher^2^	227 / 18,534 (1.2%)	209 / 15,489 (1.4%)	18 / 3,045 (0.59%)

^1^Median (IQR); n / N (%);^2^″other” includes: haemophilia, blood transfusion, occupational exposure, pre-/perinatal infection and other transmission routes not further specified

The median observation time was 9 months (interquartile range (IQR): 6–39 months) and differed between the participants of the two cohort studies. While ClinSurv-HIV participants included in this analysis had a median observation time of 8 months (IQR: 6–32 months), HIV-1 Seroconverter participants had a median observation time of 24 months (IQR: 8–58 months). The median observation time did not differ between study participants with or without records of HIV-ICs and was 9 months (IQR participants with HIV-IC: 6–42 months; IQR participants without HIV-IC: 6–31 months). Overall, 51% of participants were reported with any of the selected HIV-ICs, while 29% of participants had one diagnosed HIV-IC, 13% had two HIV-ICs and 5.5% three HIV-ICs. The remaining 3.2% of participants had 4–13 HIV-ICs.

In the following paragraphs the prevalence, incidence of the respective diagnoses will be presented in the disease categories STIs, viral hepatitis, HIV-associated and AIDS-defining diagnoses.

### Sexually transmitted infections

The prevalence of STIs among participants of the HIV-1 Seroconverter study, namely chlamydial infection, anogenital (venereal) warts, gonococcal infection, diagnoses with papillomavirus as the cause of disease and syphilis, ranged from 0.17% for diagnoses with papillomavirus as the cause of disease to 7.8% for syphilis. Similarly, the lowest incidence rate was found for papillomavirus as the cause of diseases with 1.0 per 1,000 person-years and the highest incidence for syphilis with 34 per 1,000 person-years. Prevalence and incidence among HIV-1 Seroconverter cohort participants for the respective infections is shown in Table 3. There were no diagnoses for trichomoniasis reported for the study participants, hence trichomoniasis is not included in the subsequent tables and figures.

**Table 3:** Prevalence and incidence rates among HIV-1 Seroconverter cohort participants for selected sexually transmitted infections, 2008–2023

HIV indicator condition	Prevalence in % (n/N)	Incidence rate per 1,000 PYs (95% CI)	Number of cases	Person years
Anogenital (venereal) warts	4.5% (109 / 2,422)	25 (21 – 31)	99	3,867
Chlamydial infection	3.0% (72 / 2,422)	15 (11 – 19)	58	3,880
Gonococcal infection	4.4% (107 / 2,422)	24 (19 – 29)	93	3,877
Papillomavirus as the cause of diseases	0.17% (4 / 2,422)	1.0 (0.39—2.8)	4	3,878
Syphilis	7.8% (188 / 2,422)	33 (28 – 40)	130	3,848

Considering the HIV-transmission risk of participants, there was no association with diagnosis of anogenital (veneral) warts, chlamydial infection or papillomavirus as the cause of diseases. For gonococcal infection and syphilis an association with HIV-transmission risk was found (p-value: 0.017 and 0.018, respectively). Of participants indicating MSM-contact as their HIV-transmission risk 5.0% and 8.5% tested positive for gonococcal infection or syphilis, respectively (see supplemental Table 2 for details).

The incidence rates for the STI diagnoses were determined for four different time periods, starting in 2008 as described in the methods. Incidence rates for papillomavirus were not determined due to sparse data. Starting from the first time period (2008–2010) an increasing incidence rate can be seen for all four STI analysed until the time period 2015–2018. This increase over time was confirmed by a test for trend (nogenital warts: p-value: 0.004; hlamydial infection: p-value: < 0.001; onococcal infection: p-value: < 0.001; syphilis: p-value: < 0.001). The last time period has wider confidence intervals due to the decreasing number of PYs contributed, making the interpretation of the incidence rates less reliable. An overview of the incidence rate per time period and STI is shown in Fig. 1.

### Viral hepatitis diagnoses

For the analysis of viral hepatitis, diagnoses of acute and chronic hepatitis of the types A, B, C, D, E and with unknown hepatitis type were included. Data from both cohort studies were analysed, from 1999 until 2018 for ClinSurv-HIV participants and until 2023 for HIV-1 Seroconverter participants. The analysis does not differentiate between acute and chronic hepatitis. The prevalence found for the overall study period ranged from 0.01% for hepatitis E to 11% for hepatitis B and incidence rate per 1.000 PYs ranged from 0.03 for hepatitis E to 18 for hepatitis B. Detailed information on prevalence and incidence are shown in Table 4. There were no diagnoses for hepatitis D reported.

**Table 4:** Prevalence and incidence rates among ClinSurv-HIV and HIV-1 Seroconverter cohort participants for viral hepatitis type A, B, C, E and type unknown, 1999–2023

HIV indicator condition	Prevalence in % (n/N)	Incidence rate per 1,000 PYs (95% CI)	Number of cases	Person years
Hepatitis A	6.8% (1,263 / 18,534)	8.4 (7.5 – 9.5)	256	30,392
Hepatitis B	11% (2,103 / 18,534)	18 (16 – 19)	533	29,229
Hepatitis C	5.3% (986 / 18,534)	10 (9.0 – 11)	326	32,522
Hepatitis E	0.01% (2 / 18,534)	0.03 (0.00 – 0.22)	1	32,698
Viral hepatitis (type unknown)	1.3% (247 / 18,534)	3.0 (2.4–3.6)	96	32,256

For viral hepatitis diagnoses an association with the HIV-transmission risk of participants was found for hepatitis A, B, C and viral hepatitis with unknown type (p-value < 0.001, respectively). The highest proportion of participants with hepatitis A diagnoses was found among participants reporting coming from a high-prevalence country for HIV (19%) as their likely HIV-transmission risk, followed by those reporting IDU (11%). For hepatitis B, 20% of participants reporting IDU and 20% of participant coming from a high-prevalence country for HIV had a hepatitis B diagnosis. For hepatitis C and viral hepatitis with unknown type, 40% and 6.5% of participants with the HIV-transmission risk IDU had a respective HIV-IC diagnosis (see supplemental Table 2 for details).

**Fig. 2:**
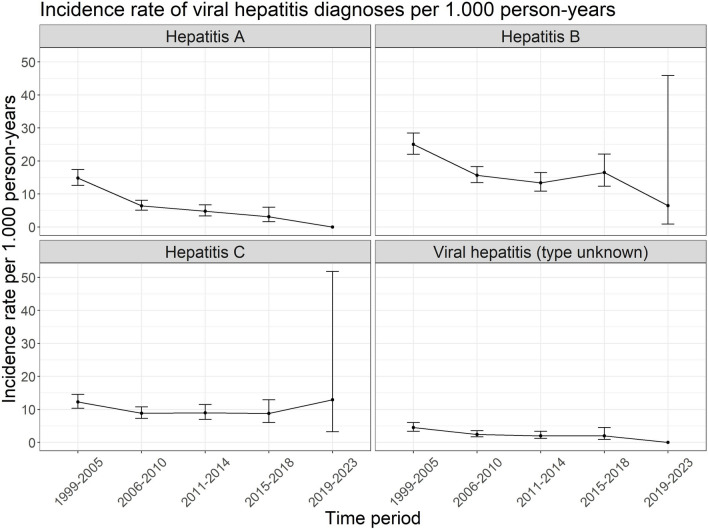
Incidence rates of viral hepatitis diagnoses per 1.000 person-years among ClinSurv-HIV and HIV-1 Seroconverter cohort participants by time period 1999–2005, 2006–2010, 2011–2014, 2015–2018 and 2019–2023

The incidence rate by time period (1999–2023) for viral hepatitis A, B, C and diagnoses with unknown type are shown in Fig. 2. Trends in diagnoses of hepatitis E were not analysed due to sparse data. There were no diagnoses found for hepatitis A and viral hepatitis (type unknown) in the time period 2019–2023. The incidence rates for hepatitis A and B decreased compared to the first time period, while the confidence intervals for hepatitis C and diagnoses with unknown type remained slightly overlapping. As mentioned for the incidence rates of STIs, the interpretation of incidence during the last time period is challenging due to the wide confidence intervals caused by the very small amount of PYs contributed. Despite these overlapping confidence intervals the test for trend showed a decreasing incidence rate for hepatitis A-C as well as hepatitis cases with unknown type (hepatitis A: p-value: < 0.001; hepatitis B: p-value: < 0.001; hepatitis C: p-value: 0.02; viral hepatitis (type unknown): p-value: < 0.001).

### HIV-associated diagnoses

We analysed the following HIV-associated diagnoses, according to the category B of the CDC-classification for HIV infections: dysplasia of the cervix uteri, herpes zoster, Hodgkin lymphoma, malignant neoplasm of anus and anal canal, malignant neoplasm of bronchus and lung, oral hairy leukoplakia, and seborrheic dermatitis. In addition, we included infectious mononucleosis to this category, as the symptoms can be very similar to those during acute HIV-infection. Again, diagnoses among participants of both cohorts were analysed from 1999–2023. No diagnoses were found for malignant neoplasm of anus and anal canal and malignant neoplasm of bronchus and lung. The lowest prevalence and incidence rates were found for Hodgkin lymphoma with 0.26% and 0.80 per 1.000 PYs, respectively. The highest prevalence and incidence rate was found for herpes zoster with 5.2% and 22 per 1.000 PYs. An overview of the prevalence and incidence rates found for the diagnoses in this category is shown in Table 5.

**Table 5:** Prevalence and incidence rates among ClinSurv-HIV and HIV-1 Seroconverter cohort participants for HIV-associated diagnoses according to category B of the CDC-classification for HIV infections, 1999–2023

HIV indicator condition	Prevalence in % (n/N)	Incidence rate per 1,000 PYs (95% CI)	Number of cases	Person years
Dysplasia of cervix uteri (women only)	1.3% (44 / 3,384)	5.5 (3.9 – 7.7)	32	5861
Herpes zoster	5.2% (966 / 18,534)	22 (20 – 24)	717	32,680
Hodgkin lymphoma	0.26% (48 / 18,534)	0.80 (0.54 – 1.2)	26	32,667
Infectious mononucleosis	2.1% (397 / 18,534)	2.1 (1.7 – 2.7)	69	32,022
Oral hairy leukoplakia	2.7% (502 / 18,534)	7.9 (7.0 – 8.9)	253	32,187
Seborrheic dermatitis	2.4% (442 / 18,534)	8.2 (7.3 – 9.2)	264	32,217

Among participants with any of the ICs considered as HIV-associated, an association with the HIV-transmission risk was found for the following diagnoses: herpes zoster (p-value: 0.02), oral hairy leucoplakia (p-value: 0.001) and seborrheic dermatitis (p-value: < 0.001). For herpes zoster, participants reporting heterosexual transmission or MSM had the highest diagnosis prevalence with 5.9% and 5.4%, respectively. For both diagnoses, oral hairy leucoplakia and seborrheic dermatitis the groups “other” and “unknown” had the highest prevalence of the respective diagnosis, while the prevalence ranged from 2.2%—2.8% and 2.1%—2.7%, respectively, for participants reporting being MSM, heterosexual transmission or IDU as their likely HIV-transmission route (see supplemental Table 2).

**Fig. 3:**
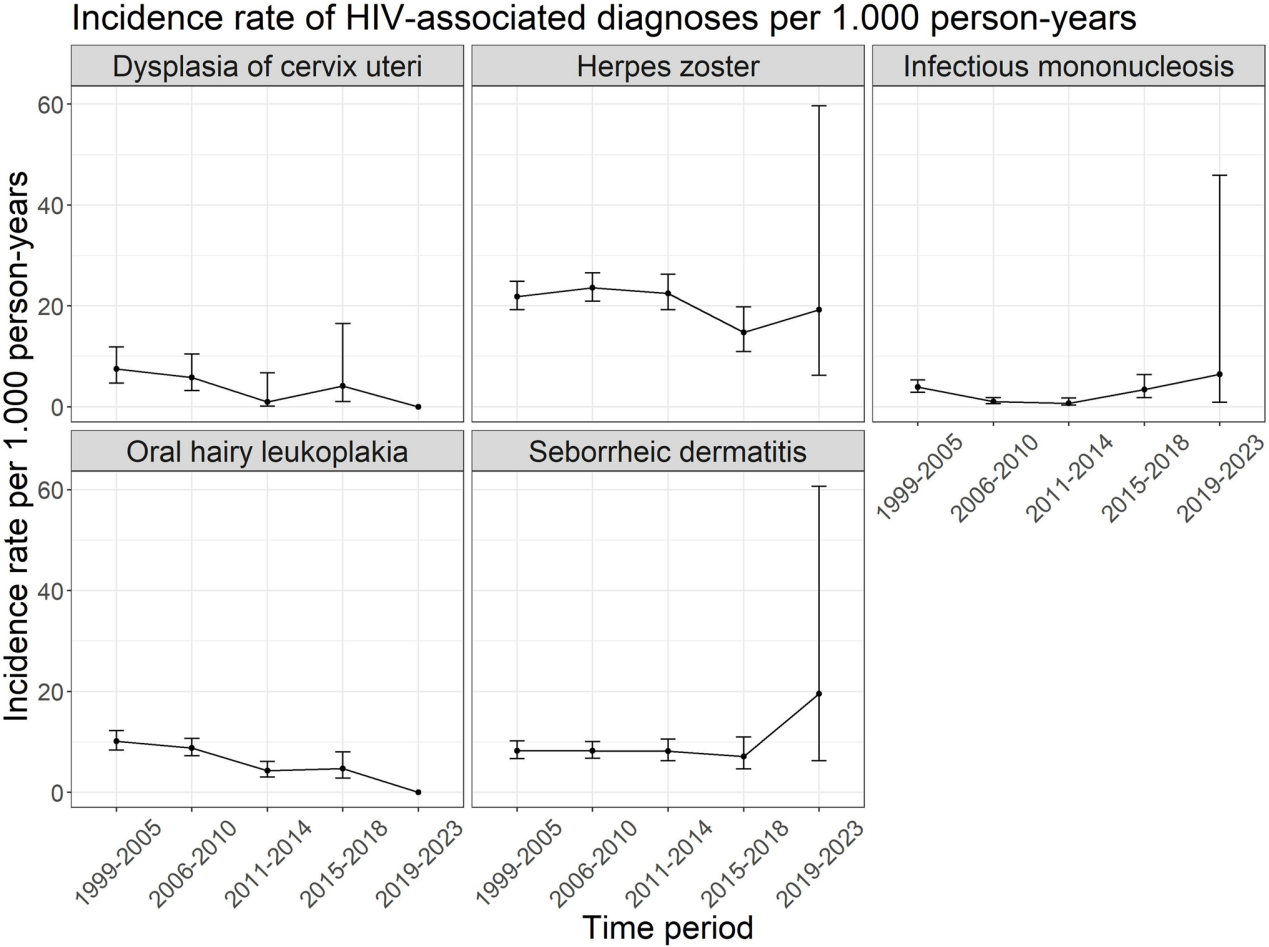
Incidence rates of HIV-associated diagnoses per 1.000 person-years among ClinSurv-HIV and HIV-1 Seroconverter cohort participants by time period 1999–2005, 2006–2010, 2011–2014, 2015–2018 and 2019–2023

The incidence rates by time period (1999–2023) and per HIV-associated diagnoses are shown in Fig. 3. There were no diagnoses found for dysplasia of cervix uteri and oral hairy leukoplakia in the time period 2019–2023. Based on the test for trend decreasing incidence rates over time can be seen for oral hairy leukoplakia (p-value: < 0.001), dysplasia of cervix uteri (p-value: 0.04) and infectious mononucleosis (p-value: 0.002). There was no change of incidence confirmed over time for herpes zoster and seborrheic dermatitis.

### AIDS-defining diagnoses

AIDS-defining diagnoses were analysed from 1999–2023. The respective diagnoses used are listed in Table 1. The highest prevalence and incidence rate found in this group of diagnoses was for candidiasis with a prevalence of 13% and an incidence rate of 30 per 1,000 PYs. Diagnosis of malignant neoplasm of cervix uteri showed the lowest prevalence with 0.2% and lowest incidence rate with 1.0 per 1,000 PYs (see Table 6).

**Table 6:** Prevalence and incidence rates among ClinSurv-HIV and HIV-1 Seroconverter cohort participants for AIDS-defining diagnoses according to category C of the CDC-classification for HIV infections, 1999–2023

HIV indicator condition	Prevalence in % (n/N)	Incidence rate per 1,000 PYs (95% CI)	Number of diagnoses	Person years
Abnormal weight loss	1.0% (184 / 18,534)	2.6 (2.1 – 3.3)	86	32,516
Candidiasis	13% (2,361 / 18,534)	30 (29 – 32)	990	32,674
Herpes simplex infections	3.6% (669 / 18,534)	14 (13 – 15)	450	31,823
Kaposi sarcoma	2.5% (455 / 18,534)	6.3 (5.5 – 7.2)	204	32,420
Malignant neoplasm of cervix uteri (women only)	0.18% (6 / 3,384)	1.0 (0.5 – 2.3)	6	5904
Non-Hodgkin lymphoma	1.2% (230 / 18,534)	3.6 (3.0 – 4.3)	116	32,567
Pneumocystosis	4.8% (897 / 18,534)	6.9 (6.1 – 7.9)	222	32,167
Pneumonia	2.9% (543 / 18,534)	10 (9.1 – 11)	330	32,699
Tuberculosis	1.8% (329 / 18,534)	4.5 (3.8 – 5.3)	145	32,413

For the AIDS-defining diagnoses an association was found between diagnosis and HIV-transmission risk for herpes simplex infection (p-value: 0.016), candidiasis (p-value: < 0.001), Kaposi sarcoma (p-value: < 0.001), non-Hodgkin lymphoma (p-value: 0.005), pneumonia (p-value: < 0.001), pneumocystis (p-value: < 0.001) and tuberculosis (p-value: < 0.001; see supplemental Table 2). Highest prevalence for herpes simplex infections was found among participants reporting heterosexual transmission (4.0%) and MSM (3.8%), for candidiasis highest prevalence was found for heterosexual transmission (15%) and IDU (14%). For Kaposi sarcoma diagnosis, people reporting MSM as their likely HIV-transmission route had the highest diagnosis prevalence (3.3%), for pneumonia diagnosis it was IDU (7.1%). The HIV-transmission route with the highest diagnosis prevalence of pneumocystis were people reporting heterosexual transmission (5.8%) and for tuberculosis the highest diagnosis prevalence was found among participant reporting coming from a country of high HIV-prevalence (6.7%).

**Fig. 4:**
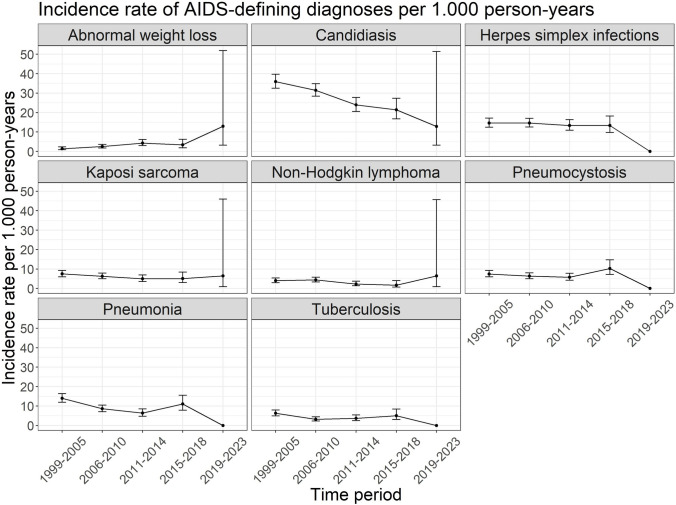
Incidence rates of AIDS-defining diagnoses per 1.000 person-years among ClinSurv-HIV and HIV-1 Seroconverter cohort participants by time period 1999–2005, 2006–2010, 2011–2014, 2015–2018 and 2019–2023

The incidence rates by time period (1999–2023) and per AIDS-associated diagnoses are shown in Fig. 4. There were no diagnoses found for herpes simplex infections, pneumocystosis, pneumonia and tuberculosis in the time period 2019–2023. Decreasing incidence rates over time, based on the test for trend, are observed for candidiasis (p-value: < 0.001), Kaposi sarcoma (p-value: 0.03), non-Hodgkin lymphoma (p-value: 0.03), pneumonia (p-value: < 0.001) and tuberculosis (p-value: 0.02). The incidence of abnormal weight loss increased over time (p-value: < 0.001).

### Further characterization of HIV-ICs

**Fig. 5:**
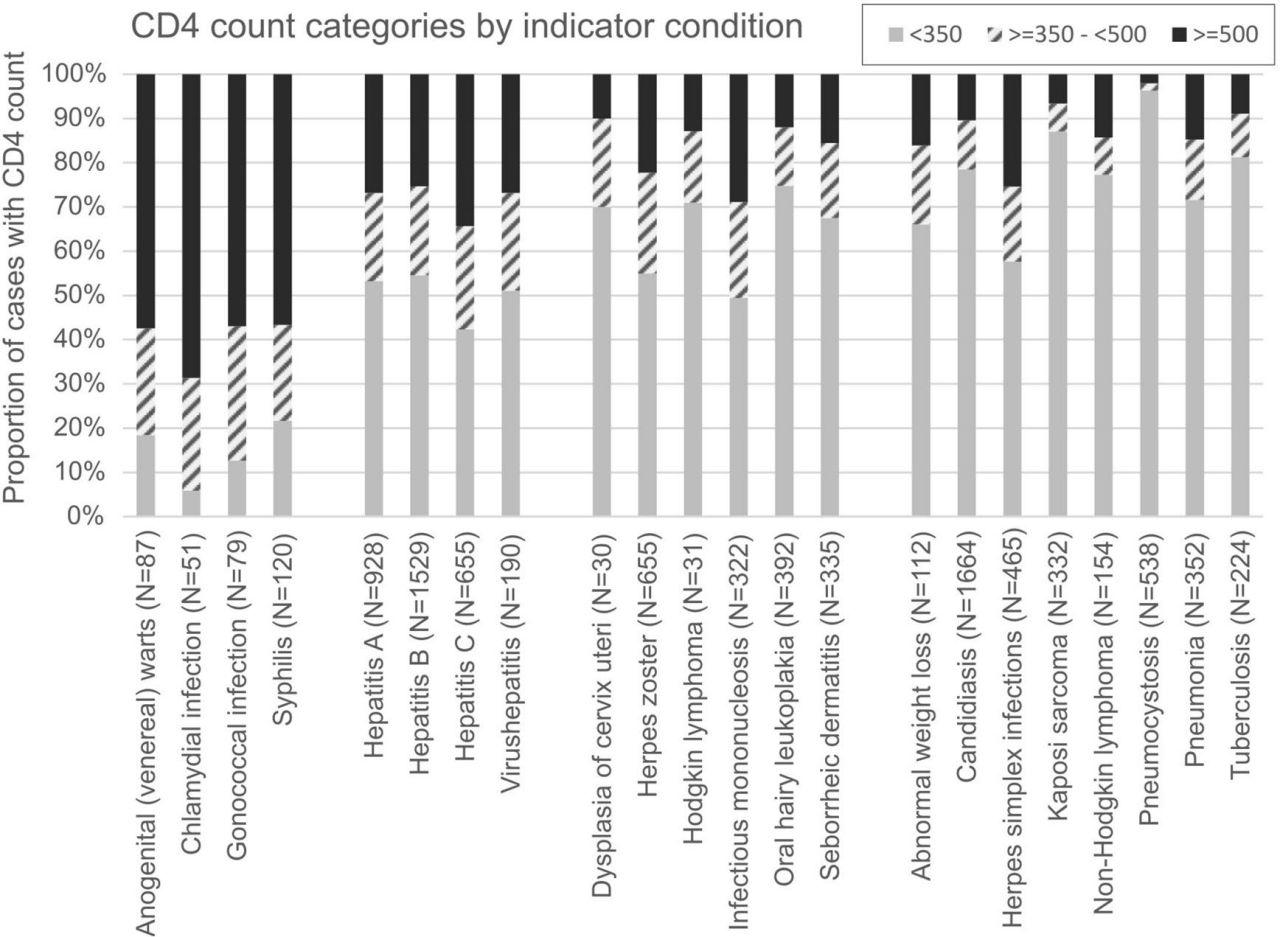
Proportion of CD4-cell count/µl blood category for diagnoses of the respective HIV-IC with available data. HIV-ICs with less than 10 available observations were excluded

To characterize the immune status, CD4 counts were analysed as a proxy. We chose the time wise closest CD4-count to the respective diagnosis (until ± 30 days) and categorised the CD4-counts into the three categories < 350 cells/µl blood, >  = 350—< 500 cells/µl blood, >  = 500 cells/µl blood. The number of diagnoses with available CD4 counts is shown in Fig. 5, while the total number of respective diagnoses can be found as the prevalence in the respective tables per disease category above. The proportion of CD4 counts is therefore based on the total number of CD4 counts available per diagnosis. Among STI diagnoses more than half of the diagnoses occurred when participants had CD4 counts of >  = 500 cells/µl, while only 5.9–22% had CD4 counts of < 350 cells/µl blood (Fig. 5). This differs for viral hepatitis diagnoses. Here 25–34% of diagnoses occurred when participants had CD4 counts of >  = 500 cells/µl blood around their hepatitis diagnosis and 42–55% CD4 counts of < 350 cells/µl. The CD4 counts at diagnosis further decrease if measured around the diagnoses of AIDS-defining conditions, such as Kaposi sarcoma, where 87% of participants have a CD4 count of < 350 cells/µl blood or pneumocystosis with 96% of participants with a CD4 count of < 350 cells/µl blood. For a detailed overview per HIV-IC, see Fig. 5.

## Discussion

The results of this analysis provide valuable insights into the diagnosis of selected HIV-ICs among a cohort of PLWH before or during the early phase of ART. Further characterization of HIV-ICs based on patients’ likely route of HIV transmission and their CD4 count—serving as a proxy for the immune status – allows to inform and help implementing HIV-IC -based testing in Germany and prevent late diagnosis.

The HIV-ICs with the highest prevalence seen for the study period were candidiasis and hepatitis B diagnoses. The highest incidence rate was seen for syphilis with an incidence rate of 34 per 1,000 PY, IQR 29–40, while candidiasis also had a high incidence rate of 30 per 1,000 PYs (IQR 29–32). Interestingly, some diagnoses were associated with the reported route of HIV-transmission, further identifying potential groups at particular risk for HIV-infections. While for example the highest diagnosis prevalence of gonococcal infection and syphilis was found among study participants reporting MSM as their likely route of HIV-transmission, the highest prevalence of hepatitis B diagnosis was found among participants coming from a high-prevalence country for HIV or reporting IDU. PWID were also the group of people with the highest prevalence of hepatitis C and pneumonia diagnosis. The immune status, based on CD4 counts measured close to the diagnosis, was worse among participants with AIDS-defining conditions. Although participants diagnosed with STIs had the highest CD4 counts, for almost half them the value was still below 500 cells/µl blood. Participants diagnosed with hepatitis or HIV-associated diagnoses had CD4 counts indicating impaired immune statuses.

The study population included in these two cohort studies and eligible for this analysis is overall comparable to PLWH in Germany with regards to characteristics such as the likely route of HIV transmission and sex distribution [4]. However, looking at the two cohort studies separately, the observation time contributed by participants until the initiation of ART differed. This is not surprising when considering the inclusion criteria of these two cohorts. While the HIV-1 Seroconverter cohort study includes participants with a known or estimated date of HIV seroconversion in order to characterize the course of HIV-infection, the ClinSurv-HIV cohort also recruits participants with an unknown HIV infection date in order to characterize the influence of therapy on disease progression [24]. Especially during time periods with delayed ART treatment recommendation based on the CD4 counts, this results in overall longer observation times among HIV-1 Seroconverter participants. Since the majority of HIV-1 Seroconverter participants reported being MSM as their likely HIV transmission route, this leads to a higher representation of MSM with regards to the observation time in this analysis. Also, recent arrival of refugees, for example from Ukraine, or other patterns of migration are not well represented by the study population for the time from 2019 and onwards. During this time period the analysis includes only results from the HIV-1 Seroconverter study. However, due to the inclusion criteria, previously diagnosed patients can often not be included. Thus, the results during this time period cannot be extrapolated to people living with HIV, who migrated to Germany during this time.

For the analysis of STI diagnoses only participants of the HIV-1 Seroconverter study were included, where 87% of participants reported being MSM. This limits the representativeness and ability of this analysis to further characterize STIs as HIV-ICs for patients with other routes of HIV transmission. It is also important to keep in mind different screening frequencies for STIs when included in these clinically well supervised cohort studies. This may however be particularly the case for diagnoses that are typically screened for by serum samples (e.g. syphilis) as opposed to those needing physical examination (anogenital warts). In addition to potentially different sexual risk behaviour in this group of patients, this might also further contribute to the 180 × higher incidence found in this analysis for syphilis (incidence rate 34 per 1000 PYs for 2008–2023) compared to the incidence of notified syphilis cases in 2022 in the general male population with for example 19 cases per 100,000 inhabitants [25]. Comparing with users of HIV pre-exposure prophylaxis, among whom 99% identify as MSM, a three times higher incidence of 100/1000 PYs was found [26]. When looking at the self-reported prevalence of syphilis among MSM with 3%, the syphilis prevalence found in our analysis among a group of PLWH, mostly reporting MSM as their likely risk of HIV transmission, is still more than double [27]. STIs have been confirmed in other studies as HIV-ICs and also with the first HeLP study part, analysing HIV prevalence among patients with the respective STIs based on statutory health insurance data, we confirmed a HIV prevalence > 0.1% in Germany for the here examined STIs [12, 17, 28]. The similar transmission route of STIs and HIV might also allow an early detection of HIV infection when considered as HIV-ICs and hence screened for after STI diagnosis. This is already considered in the respective treatment guidelines for example for gonococcal infection, syphilis and—for people with respective risk behaviour—the more unspecific guideline for STI diagnosis, where an HIV test is recommended upon respective STI diagnosis [29–31]. Testing patients with STI diagnoses would allow the early initiation of ART, when patients still have a good immune status, with the resulting health benefits for PLWH [6, 32]. This is also supported by our results, where we see overall higher CD4 counts (> = 500 cells/µl blood) among the majority of participants diagnosed with STIs compared to those diagnosed with other HIV-ICs, but at the same time a substantial amount of participants with STIs and even more participants with other diagnoses who show signs of an impaired immune system.

With regards to viral hepatitis diagnoses this analysis finds increased incidence rates for hepatitis A, hepatitis B and hepatitis C diagnoses compared to the mandatory notification incidence of the respective hepatitis virus infections in Germany [33]. Also the hepatitis B and C prevalence is increased compared to the self-reported prevalence among MSM, while the prevalence found for hepatitis A is similar [27]. Although this study population is not representative of the general population in Germany, reported HIV transmission paths among participants testing positive well reflect the transmission paths of mandatory notifications for the respective hepatitis types (high proportion of IDU transmission for hepatitis B and C diagnoses) [33]. Vaccination recommendations for hepatitis B virus is likely to have contributed to the reduction of hepatitis B incidence over the study period, however still remaining with increased incidence rates [33, 34]. Reports of hepatitis A cases and outbreaks among MSM also increase the role of sexual transmission for hepatitis A virus infections and therefore making hepatitis A an important HIV-IC [35, 36]. Viral hepatitis diagnoses have also been confirmed as an HIV-IC in a number of studies [12, 28], likewise by the statutory health insurance data analysis of the HeLP study [17].

HIV-associated ICs include also sequelae of infections or malignant disease. In our analysis for example herpes zoster had the highest incidence. Other studies found less explicit results for the HIV prevalence among patients diagnosed with herpes zoster, some confirming its diagnosis as an HIV-IC [12, 37, 38], others find HIV prevalences < 0.1% overall or for example for women or certain age-groups [17, 28, 39]. Some of these studies are limited though in their representativeness due to the low number of people investigated [39]. Potentially decreasing incidence rates over time of HIV-ICs that are associated with being immunocompromised, such as oral hairy leucoplakia, is likely explained by the changes in recommendation for earlier initiation of ART and therefore reduced likelihood of disease development [40]. This does not allow conclusions on the importance of the respective diagnosis as an HIV-IC but is a limitation of this analysis, which includes participants with a known HIV diagnosis who are well medically supervised and might therefore have decreasing incidence rates for the respective diagnoses. The analysis of CD4 counts confirms reduced CD4 counts for participants with diagnoses that are associated with being immunocompromised such as oral hairy leucoplakia or seborrheic dermatitis and highlights the importance to offer HIV testing to patients with these diagnoses in order to detect undiagnosed PLWH who might already be immunocompromised.

AIDS-defining conditions are HIV-ICs by definition [41]. In this analysis we find surprisingly high incidence rates for candidiasis and likely overestimate the incidence as an AIDS-defining condition. This might be caused by the rather unspecific diagnosis code included in this analysis, which is “candidiasis” without further specification of the location, while only “Candidiasis of bronchi, trachea, or lungs” and “Candidiasis of esophagus” are AIDS-defining conditions [41]. It may also be the case for pneumonia. While only recurring pneumonia is defined as an AIDS-defining condition, we included all reported pneumonia diagnoses due to the structure of the data available [41]. Nevertheless and compared across the HIV-ICs analysed, participants diagnosed with AIDS-defining conditions tend to have the lowest CD4 counts with almost three quarter of participants with less than 350 cells/µl. With regards to the HIV transmission routes, the highest proportion of participants with tuberculosis diagnosis reported coming from an HIV-high-prevalence country. This is likely explained by the overlap of countries with high HIV and tuberculosis prevalence [42].

This study has a number of limitations that might impact the generalizability of the results. The data for the analyses are derived from people living with diagnosed HIV infections and under clinical care. It is unclear to what extent the results can be extrapolated to undiagnosed persons with HIV, e.g. HIV-ICs might also occur at later stages with more impaired immune status. Therefore, an analysis of HIV prevalence in the general population has been conducted based on statutory health insurance data in the first part of this project [16, 17]. In addition, the completeness of the diagnoses reported in these two cohort studies is uncertain due to the way of reporting. The ClinSurv-HIV study relies on unsupported reporting, which might result in underreporting especially of diseases that are not directly associated with HIV. The overall observation period of participants until the initiation of ART can be rather short and therefore does not allow for longer time periods for the occurrence and diagnoses of HIV-ICs. This might lead to an underreporting. At the same time study participants are likely to be medically better supervised, which on the contrary might result in increased diagnoses of certain HIV-ICs due to better awareness of treating physicians. Especially during the last time period for incidence analysis (2019–2023), the COVID-19 pandemic and possibly less frequent doctoral visits may have resulted in underdiagnosis and underreporting of indicator conditions. Last but not least the diagnosis of HIV-ICs is based on ICD-10 codes, which leads to less specified analysis of some conditions (e.g. for pneumocystis the ICD codes do not differentiate between the infection and the disease or for candidiasis very general codes were chosen).

The strength of the study is however the availability of CD4 counts close to the diagnosis of respective HIV-ICs as a proxy for the immune status. Since we only included participants before the initiation of ART or during the early phase of ART this allows insights into the immune status when people are diagnosed with an HIV-IC and are also HIV-positive.

## Conclusion

This analysis presents important findings that allow the further characterization of HIV-ICs with regards to the HIV transmission route and the immune status of patients diagnosed. It also allows the analysis of the incidence of HIV-ICs in the largest available population of PLWH over more than 20 years.

The results underline the importance of HIV-IC-based screening and the opportunity to detect patients early and with a good immune status. Nevertheless, HIV screening based on ICs has so far been poorly implemented in many European countries or even for selected diagnoses that would even need to be considered with a risk-based screening approach [15, 43]. An approach with electronic alerts as a reminder for HIV testing when any of the confirmed HIV-ICs are diagnosed could be helpful [44].

## Supplementary Information

Below is the link to the electronic supplementary material.

Supplementary file1 (JPG 274 KB)

Supplementary file2 (DOCX 24 KB)

## Data Availability

The data generated and/or analysed during the current study are not publicly available due to data protection and confidentiality agreements.
