# Supplementary figures and images for: Characterising HIV-Indicator conditions among two nationwide long-term cohorts of people living with HIV in Germany (1999–2023)
Source: Infection. 2024 Oct 30;53(3):1013–28. doi: 10.1007/s15010-024-02419-2 (PMC12137405; doi:10.1007/s15010-024-02419-2)

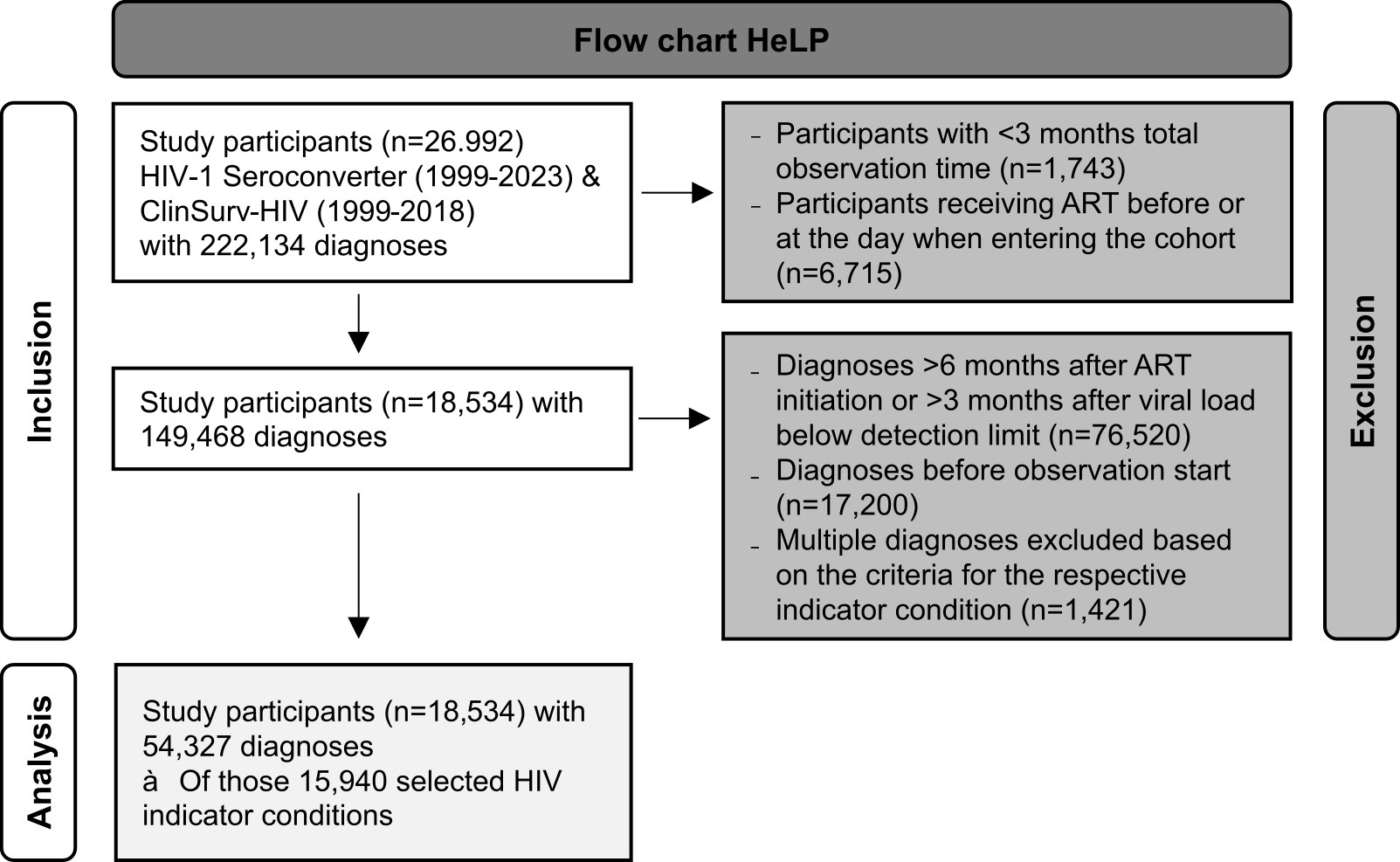

Supplement: Supplementary file 1 — Supplementary file1 (JPG 274 KB) [file 15010_2024_2419_MOESM1_ESM.jpg]
