# Supplementary material for: Characterising HIV-Indicator conditions among two nationwide long-term cohorts of people living with HIV in Germany (1999–2023)
Source: Infection. 2024 Oct 30;53(3):1013–28. doi: 10.1007/s15010-024-02419-2 (PMC12137405; doi:10.1007/s15010-024-02419-2)
Supplement: Supplementary file 2 — Supplementary file2 (DOCX 24 KB) [file 15010_2024_2419_MOESM2_ESM.docx]

**Supplement (Tables)**

Supplemental Table 1: Incidence rates and 95% Cis per 1,000 PYs by time episode.

| HIV Indicator Condition | 1999-2005 | 2006-2010  (for STIs 2008-2010) | 2011-2014 | 2015-2018 | ab 2019 |
| --- | --- | --- | --- | --- | --- |
| *Sexually transmitted infections* |  |  |  |  |  |
| Anogenital (venereal) warts | n/a | 18 (13 - 26) | 27 (20 - 36) | 39 (26 - 60) | 39 (17 - 86) |
| Chlamydial infection | n/a | 6.4 (3.5 - 12) | 16 (11 - 24) | 32 (20 - 51) | 26 (9.7 - 69) |
| Gonococcal infection | n/a | 9.0 (5.3 - 15) | 19 (14 - 28) | 64 (46 - 89) | 78 (44 - 137) |
| Syphilis | n/a | 16 (11 - 24) | 35 (27 - 46) | 71 (52 - 98) | 67 (36 - 125) |
| *Viral Hepatitis infections* |  |  |  |  |  |
| Hepatitis A | 15 (13 - 17) | 6.4 (5.1 - 8.1) | 4.8 (3.4 - 6.7) | 3.1 (1.6 - 6.0) | n/a |
| Hepatitis B | 25 (22 - 28) | 16 (13 - 18) | 13 (11 - 17) | 17 (12 - 22) | 6.5 (0.91 - 46) |
| Hepatitis C | 12 (10 - 15) | 8.9 (7.3 - 11) | 9.0 (7.0 - 12) | 8.8 (6.0 - 13) | 13 (3.2 - 52) |
| Viral hepatitis type unknown | 4.5 (3.4 - 6.0) | 2.4 (1.7 - 3.5) | 2.0 (1.2 - 3.4) | 2.0 (0.91 - 4.5) | n/a |
| *HIV-associated diagnoses (CDC-category B)* |  |  |  |  |  |
| Dysplasia of cervix uteri | 7.5 (4.7 - 12) | 5.8 (3.2 - 11) | 0.94 (0.13 - 6.7) | 4.1 (1.0 - 17) | n/a |
| Herpes zoster | 22 (19 - 25) | 24 (21 - 27) | 23 (19 - 26) | 15 (11 - 20) | 19 (6.2 - 60) |
| Infectious mononucleosis | 3.9 (2.9 - 5.3) | 1.1 (0.59 - 1.8) | 0.71 (0.30 - 1.7) | 3.4 (1.8 - 6.3) | 6.5 (0.91 - 46) |
| Oral hairy leukoplakia | 10 (8.4 - 12) | 8.8 (7.2 - 11) | 4.3 (3.0 - 6.2) | 4.8 (2.8 - 8.0) | n/a |
| Seborrheic dermatitis | 8.3 (6.7 - 10) | 8.3 (6.8 - 10) | 8.2 (6.3 - 11) | 7.2 (4.7 - 11) | 20 (6.3 - 61) |
| *AIDS-defining diagnoses (CDC-category C)* |  |  |  |  |  |
| Abnormal weight loss | 1.4 (0.84 - 2.3) | 2.5 (1.7 - 3.6) | 4.3 (3.0 - 6.1) | 3.4 (1.8 - 6.3) | 13 (3.2 - 52) |
| Candidiasis | 36 (33 - 40) | 32 (28 - 35) | 24 (21 - 28) | 21 (17 - 27) | 13 (3.2 - 51) |
| Herpes simplex infections | 15 (13 - 17) | 15 (13 - 17) | 13 (11 - 16) | 13 (9.7 - 18) | n/a |
| Kaposi sarcoma | 7.5 (6.0 - 9.3) | 6.3 (5.0 - 7.9) | 5.0 (3.6 - 6.9) | 5.1 (3.0 - 8.4) | 6.5 (0.91 - 46) |
| Non-Hodgkin lymphoma | 4.0 (3.0 - 5.4) | 4.4 (3.3 - 5.8) | 2.3 (1.4 - 3.7) | 1.7 (0.7 - 4.0) | 6.4 (0.91 - 46) |
| Pneumocystosis | 7.5 (6.0 - 9.3) | 6.3 (5.0 - 8.0) | 5.7 (4.2 - 7.8) | 10 (7.2 - 15) | n/a |
| Pneumonia | 14 (12 - 16) | 8.7 (7.1 - 11) | 6.4 (4.8 - 8.5) | 11 (7.8 - 16) | n/a |
| Tuberculosis | 6.3 (4.9 - 8.0) | 3.2 (2.3 - 4.4) | 3.7 (2.5 - 5.4) | 5.1 (3.1 - 8.4) | n/a |

Supplemental Table 2: Proportion and number of participants testing positive for the respective HIV-IC by reported HIV-transmission route as well as p-value based on Pearson’s Chi^2^ test result.

| Indicator condition | MSM (N=2,128) | IDU (N=34) | Heterosexual (N=186) | HPC (N=13) | other (N=14) | unknown (N=47) | Total (N=2,422) | p-value |
| --- | --- | --- | --- | --- | --- | --- | --- | --- |
| *Sexually transmitted infections* |  |  |  |  |  |  |  |  |
| Anogenital (venereal) warts | 4.7% (100) | 0.0% (0) | 4.3% (8) | 0.0% (0) | 0.0% (0) | 2.1% (1) | 109 | 0.593 |
| Chlamydial infection | 3.4% (72) | 0.0% (0) | 0.0% (0) | 0.0% (0) | 0.0% (0) | 0.0% (0) | 72 | 0.068 |
| Gonococcal infection | 5.0% (106) | 0.0% (0) | 0.54% (1) | 0.0% (0) | 0.0% (0) | 0.0% (0) | 107 | 0.021 |
| Syphilis | 8.5% (180) | 0.0% (0) | 4.3% (8) | 0.0% (0) | 0.0% (0) | 0.0% (0) | 188 | 0.018 |
| Indicator condition | **MSM (N=10,352)** | **IDU (N=1,318)** | **Heterosexual (N=2,964)** | **HPC (N=1,854)** | **other (N=227)** | **unknown (N=1,819)** | **Total (N=18,534)** | **p-value** |
| *Viral Hepatitis infections* |  |  |  |  |  |  |  |  |
| Hepatitis A | 4.4% (453) | 11% (142) | 6.5% (193) | 19% (345) | 4.0% (9) | 6.7% (121) | 1,263 | 0.000 |
| Hepatitis B | 10% (1,044) | 20% (268) | 8.3% (246) | 20% (368) | 8.4% (19) | 8.7% (158) | 2,103 | 0.000 |
| Hepatitis C | 2.5% (261) | 40% (532) | 3.1% (91) | 0.92% (17) | 7.5% (17) | 3.7% (68) | 986 | 0.000 |
| Viral hepatitis type unknown | 0.72% (75) | 6.5% (85) | 1.0% (30) | 1.5% (28) | 4.4% (10) | 1.0% (19) | 247 | 0.000 |
| *HIV-associated diagnoses (CDC-category B)* |  |  |  |  |  |  |  |  |
| Herpes zoster | 5.4% (558) | 3.6% (47) | 5.9% (176) | 4.8% (88) | 4.0% (9) | 4.8% (88) | 966 | 0.022 |
| Hodgkin lymphoma | 0.31% (32) | 0.15% (2) | 0.13% (4) | 0.16% (3) | 0.0% (0) | 0.38% (7) | 48 | 0.332 |
| Infectious mononucleosis | 2.2% (228) | 1.7% (22) | 1.7% (49) | 2.8% (51) | 0.88% (2) | 2.5% (45) | 397 | 0.053 |
| Oral hairy leukoplakia | 2.8% (286) | 2.2% (29) | 2.9% (86) | 1.5% (27) | 5.3% (12) | 3.4% (62) | 502 | 0.001 |
| Seborrheic dermatitis | 2.7% (274) | 2.1% (27) | 2.4% (71) | 0.7% (12) | 3.1% (7) | 2.8% (51) | 442 | 0.001 |
| *AIDS-defining diagnoses (CDC-category C)* |  |  |  |  |  |  |  |  |
| Abnormal weight loss | 1.0% (108) | 0.83% (11) | 1.1% (32) | 0.43% (8) | 1.3% (3) | 1.2% (22) | 184 | 0.162 |
| Candidiasis | 12% (1,212) | 14% (183) | 15% (445) | 11% (200) | 12% (28) | 16% (293) | 2,361 | 0.000 |
| Herpes simplex infections | 3.8% (398) | 2.1% (28) | 4.0% (118) | 3.4% (63) | 2.2% (5) | 3.1% (57) | 669 | 0.016 |
| Kaposi sarcoma* | 3.3% (336) | 0.30% (4) | 1.3% (37) | 1.5% (28) | 0.0% (0) | 2.8% (50) | 445 | 0.000 |
| Non-Hodgkin lymphoma | 1.2% (127) | 0.68% (9) | 1.3% (38) | 0.81% (15) | 1.3% (3) | 2.1% (38) | 230 | 0.005 |
| Pneumocystosis | 4.2% (431) | 3.3% (43) | 5.8% (173) | 3.6% (67) | 4.9% (11) | 9.5% (172) | 897 | 0.000 |
| Pneumonia | 2.0% (208) | 7.1% (93) | 3.2% (94) | 3.9% (73) | 2.6% (6) | 3.8% (69) | 543 | 0.000 |
| Tuberculosis* | 0.67% (69) | 2.2% (29) | 2.0% (58) | 6.7% (125) | 0.88% (2) | 2.5% (46) | 329 | 0.000 |

*** Pearson’s chi-squared test was used since the Fisher’s exact test could not be computed.**
